# “2.5 g, I could do that before noon”: a qualitative study on people who use drugs’ perspectives on the impacts of British Columbia’s decriminalization of illegal drugs threshold limit

**DOI:** 10.1186/s13011-023-00547-w

**Published:** 2023-06-15

**Authors:** Farihah Ali, Cayley Russell, Alissa Greer, Matthew Bonn, Daniel Werb, Jürgen Rehm

**Affiliations:** 1 Centre for Addiction and Mental Health (CAMH), Institute for Mental Health Policy Research, 33 Usrula Frank St., Toronto, ON M5S 2S1 Canada; 2 Ontario Node, Canadian Research Initiative in Substance Misuse (CRISM), 33 Ursula Franklin St., Toronto, ON M5S 2S1 Canada; 3 School of Criminology, Simon Fraser University, 8888 University Drive, Burnaby, BC V5A 1S6 Canada; 4 Canadian Association of People Who Use Drugs, 102-68 Highfield Park Drive, Dartmouth, NS B3A 1X4 Canada; 5 Centre On Drug Policy Evaluation, Unity Health Toronto, 209 Victoria St, Toronto, ON Canada; 6 Institute of Health Policy, Management and Evaluation, University of Toronto, Toronto, ON Canada; 7 Division of Infectious Diseases and Global Public Health, University of California San Diego School of Medicine, 9500 Gilman Dr., La Jolla, CA USA; 8 Department of PsychiatryDalla Lana School of Public Health, &Institute of Medical Science (IMS), University of Toronto, 1 King’s College Circle, Toronto, ON M5S 1A8 Canada; 9 Campbell Family Mental Health Research Institute, Centre for Addiction and Mental Health (CAMH), 1001 Queen St. West, Toronto, ON M6J 1H4 Canada; 10 Institut Für Klinische Psychologie Und Psychotherapie, Technische Universität Dresden, Chemnitzer Str. 46, 01187 Dresden, Germany; 11 Center for Interdisciplinary Addiction Research (ZIS), Department of Psychiatry and Psychotherapy, University Medical Center Hamburg-Eppendorf (UKE), Martinistraße 52, 20246 Hamburg, Germany

**Keywords:** Opioids, Canada, Drug Policy, Decriminalization, Public Health, Threshold Quantity

## Abstract

**Background:**

In May 2022, Health Canada approved a three-year exemption from the Controlled Drugs and Substances Act decriminalizing possession of certain illegal substances for personal use among adults in the province of British Columbia. The exemption explicitly includes a cumulative threshold of 2.5 g of opioids, cocaine, methamphetamine, and MDMA. Threshold quantities are commonly included in decriminalization policies and justified within law enforcement systems to delineate personal use among people who use drugs versus drug dealers who are carrying for trafficking purposes. Understanding the impact of the 2.5g threshold can help define the extent to which people who use drugs will be decriminalized.

**Methods:**

From June-October 2022, 45 people who use drugs from British Columbia were interviewed to gain an understanding of their perceptions on decriminalization, particularly on the proposed threshold of 2.5 g. We conduced descriptive thematic analyses to synthesize common interview responses.

**Results:**

Results are displayed under two categories: 1) Implications for substance use profiles and purchasing patterns, including implications on the cumulative nature of the threshold and impacts on bulk purchasing, and 2) Implications of police enforcement, including distrust of police use of discretion, potential for net widening and jurisdictional discrepancies in enforcing the threshold. Results illustrate the need for the decriminalization policy to consider diversity in consumption patterns and frequency of use among people who use drugs, the inclination to purchase larger quantities of substances for reduced costs and to guarantee a safe and available supply, and the role police will play in delineating between possession for personal use or trafficking purposes.

**Conclusions:**

The findings underscore the importance of monitoring the impact of the threshold on people who use drugs and whether it is countering the goals of the policy. Consultations with people who use drugs can help policymakers understand the challenges they may face when trying to abide by this threshold.

## Background

Canada is currently battling its worst illegal drug overdose crisis in history, with the province of British Columbia (BC) experiencing one of the highest rates of overdoses in Canada, rising from 34.8/100,000 population in 2020 to 44.1/100,000 population in 2021 [1]. A lot of these overdose deaths have been attributed to an increasingly volatile and toxic drug supply contaminated with fentanyl and fentanyl analogues. More recently, in BC there has been an influx of benzodiazepine-laced opioids referred to as “Benzodope” [2]. The consequence of this combination of deadly substances has led to increasing overdose death rates. As of June 2022, data indicate that the province is on track for another potential record-breaking year, with 1,121 overdose deaths recorded since January alone [1]. In 2021, overdose deaths in BC were the leading cause of unnatural death and exceeded that of homicides, suicides, motor vehicle deaths, drownings and fire-related deaths combined [3, 4]. With overdoses continuing to rise, there is an urgent need for more comprehensive efforts to reduce drug use and related harms.

Many of the response efforts implemented to address the overdose crisis have been hindered by the ongoing criminalization of drugs. The criminalization of people who use drugs results in significant social and economic harm and creates a hostile environment to access adequate healthcare [5]. For instance, criminalization stigmatizes people who use drugs and deters them from accessing necessary health and social services, calling emergency services in the event of an overdose, and perpetuates risky drug use behaviors such as using alone [6]. Furthermore, there are long-lasting negative impacts from criminal drug laws, including criminal records which impede employment, housing, travel, and social well-being, and access to treatment can be limited or interrupted, particularly for those who cycle in and out of incarceration [6]. Moreover, substance use in BC cost an estimated $6.6 billion dollars in 2017, $1.2 billion of which was directly related to costs to the criminal justice system (e.g., policing, courts, correctional system) [7]. Based on the drawbacks of using a criminal justice framework to address the overdose crisis, a more comprehensive and evidence-based approach is needed, such as decriminalization. Decriminalization is not a single model, but rather a range of principles and policies that can be combined and tailored based on the context or resources available, and can be used to reframe and underscore the public health implications of substance use [8, 9]. One of the main goals of decriminalization is to improve access to health and social services for people who use drugs by reducing stigma and building trust, as well as to reduce the burden and cost of drug possession on the criminal justice system.

Over the past few decades, several countries, including Portugal, the United States, Mexico, and Australia, have implemented various drug decriminalization strategies, some with thresholds. Threshold quantities are commonly included in decriminalization policies and justified within law enforcement systems to delineate personal use among people who use drugs versus drug dealers who are carrying for trafficking purposes. In 2001, Portugal decriminalized the acquisition, possession, and use of “small quantities” (less than 10 days’ worth; each drug has a specific threshold) of all drugs for personal use [10]. Instead of facing criminal charges, individuals are referred to a Commission for the Dissuasion of Drug Addiction, an administrative body comprising of health, social, and legal experts who assess the individual’s circumstances to identify the best possible response [10]. This paradigm shift from criminalization to a harm reduction-based approach was accompanied by the introduction of other health and social initiatives including shelters, needle and syringe programs, and drop-in centres that facilitate and increase access to treatment.

In 2009, Mexico passed federal legislation to partially decriminalize possession of small, specified amounts of drugs for personal use, including cocaine (0.5 g), methamphetamine (< 40-mlligrams), and cannabis (< 5 g) [11, 12]. In November 2020, Oregon became the only U.S. state to decriminalize the personal use of small amounts of LSD (40 units), psilocybin (12 g), methadone (40 units), oxycodone (40 pills), heroin (1 g), MDMA (1 g or 5 pills), methamphetamine (2 g), and cocaine (2 g), with the purpose of redirecting funds to treatment instead of the criminal justice system [13, 14]. Australia has had various forms of drug decriminalization since 1987, primarily for cannabis, however, threshold quantities differ depending on the state or territory; as of 2019, all Australian states had minimally implemented de facto (in practice) decriminalization of cannabis, and in October 2023, the Australian Capital Territory will implement *de jure* (in law) decriminalization of small amounts of illicit substances [15, 16]. Many of these reforms were accompanied by expansions of healthcare resources into drug treatment and harm reduction programs, and the role of law enforcement varies (e.g., some still incorporate administrative sanctions such as fines, mandatory treatment, three-strike rules, or have integrated formal diversion pathways).

Recognizing the rising rates of overdoses and the ineffectiveness of the criminal justice approach in Canada—and in the province of BC in particular—in May 2022, the federal government granted BC an exemption under Sect. 56.1 of the Controlled Drugs and Substance Act (CDSA) to decriminalize the possession of certain illegal substances for personal possession. The ultimate goal of decriminalization is to address the overdose crisis by promoting access to healthcare and social services [17, 18]. The exemption was granted for a three-year period, starting on January 31, 2023. The policy explicitly includes a cumulative threshold of 2.5 g of opioids, cocaine, methamphetamine, and/or MDMA, where individuals found with a combined amount of these specific substances under the threshold will not be subject to criminal penalties; possession above the 2.5g threshold remains a criminal offence for both possession or trafficking [18]. The exemption does not include other commonly found drugs in BC’s unregulated drug supply, such as benzodiazepines, and includes a broad definition of trafficking as defined and still included in the CDSA, including the activities of giving away, selling, supplying, administering, transferring, transporting, or delivering drugs [18].

To help inform decision-making regarding the threshold amount, BC’s Ministry of Mental Health and Addiction (MMHA)’s Decimalization Core Planning Table conducted consultations and reviewed reports from key stakeholder groups which included research from three longitudinal cohorts of approximately 1,400 people who use drugs from Vancouver prior to January 2019 [19, 20]. Based on this research, MMHA’s exemption request suggested that the proposed threshold should be set to 4.5 g to account for several factors, particularly in relation to the diversity of people who use drugs’ substance use profiles and tolerances, including actual use patterns “whereby individuals can often possess a multi-day supply for personal use” [20]. However, many advocacy groups suggested this amount was still too low and expressed frustration with what they perceived as not being given a ‘seat at the table’ during these consultations, and suggested that the police had too large of a role in the development of the policy [21]. Other key stakeholders also publicly emphasized the importance of the threshold quantity being reflective of people who use drugs’ purchasing and use patterns, for instance, BC’s Provincial Health Officer suggested that “there is no ideal threshold for personal use and setting the amount too low will undermine the goal of decriminalization and potentially lead to risks and harms” [19]. Despite the provincial endorsement of 4.5 g, the final approved threshold defined and decided by the federal government was 2.5 g, which was more aligned with the BC Association of Chiefs of Police’s recommendation for a 1g threshold, and not based off of the recommendations of MMHA and other key stakeholders who advocated for higher thresholds [22]. This decision was criticized by many harm reduction and drug advocacy groups, such as Vancouver Area Network of Drug Users (VANDU), Canadian Drug Policy Coalition, Canadian Association of People Who Use Drugs (CAPUD), and Moms Stop the Harm, whom all expressed that the 2.5g threshold limit is indisputably not reflective of people who use drugs’ purchasing and consumption patterns, and would not address the goals of the decriminalization policy in reducing harms associated with use [23].

Thus, given the risk that the 2.5g threshold limit is not in line with the recommended threshold by MMHA, people who use drugs, and harm reduction organizations, this policy detail will likely play a significant role in whether decriminalization in BC will achieve its goals, which is ultimately contingent on its implementation and enforcement. As BC is the first province to undertake this historic drug policy reform in Canada, it is fundamental to gain an understanding of the potential implications that the threshold—which defines whether an individual will be criminalized for drug possession or not—will have on people who use drugs who are directly impacted by the policy. As such, the aim of the current study was to examine the perceptions of the decriminalization policy and, specifically, the proposed threshold limit of 2.5 g among people who use drugs in BC prior to the implementation of this policy.

## Methods

Participants were recruited from a pre-existing cohort of n = 200 people who use drugs who were initially involved in a national qualitative study conducted by the Ontario Node of the Canadian Research Initiative in Substance Misuse (CRISM), a national research network, in May 2020 [24, 25]. During that study, participants provided consent to be re-contacted for future research, as well as their contact information. Using this information, a member of our research team either called or emailed past participants specifically from BC (*n* = 42) to gauge interest in participating in the current study. As many participants in the cohort study were lost to follow-up (*n* = 30), we recruited additional participants (*n* = 33) by sharing recruitment materials with existing CRISM stakeholders and people who use drugs in BC who circulated this information throughout their networks. A total of *n* = 45 people who use drugs participated in the present study.

Potential participants contacted the study team via a study email or a toll-free dedicated study line. All interested individuals were screened for study eligibility and scheduled for an interview. Our aim was to interview as many people who use drugs until we reached data saturation (i.e., no new information was gleaned from the interviews in regards to our interview questions). While some participants identified as working within the harm reduction field, many of their responses were reflective of their own personal experiences as well as their experiences working or engaging closely with other people who use drugs in various capacities. 

### Eligibility criteria

All participants needed to meet four primary eligibility criteria to participate in the study: 1) Be aged 18 years or older; 2) Currently use illegal substances (not including cannabis, since it is legal in Canada) at least weekly or be engaged in opioid agonist treatment (OAT); 3) Currently reside in BC; and 4) Speak and comprehend English.

### Data collection

Data collection commenced following the public announcement of the decriminalization policy and occurred between June 9^th^ and October 28^th^, 2022. Interviews were conducted over the phone, were audio recorded for transcription purposes, and all participants provided informed verbal consent prior to the interview. The semi-structured interview guide was developed by research team members in extensive consultation with peer advisors that have lived/living experience of drug use. Interview questions explored people who use drugs’ perspectives on the potential impacts of decriminalization of certain illegal substances, including perceptions related to benefits, challenges, concerns, or risks, as well as in relation to the 2.5g threshold specifically. All interviews were approximately 30–60 min in length, confidential, and conducted with one of two trained members of the research team (FA and CR).

### Data analysis and synthesis

This study employed a descriptive qualitative thematic analysis approach that included identifying, analyzing, and reporting common responses to our interview questions [26]. All interview transcripts were imported into qualitative data management and analysis software (NVivo) [27] and subsequently manually reviewed by a member of the research team (FA) who identified and coded common responses. FA created an initial codebook that incorporated several overarching categories based on our research questions (e.g., perspectives on the impacts of decriminalization, perspectives on the impacts of the 2.5g threshold, and perspectives on the impacts of police enforcement including current community-level experiences). FA reviewed the data again, and smaller more nuanced categories and sub-categories were coded under these larger categories. This process was completed repeatedly until all data was coded into concrete categories, which were further synthesized and refined through the writing process. Once the final codebook was completed, an independent coder (CR) randomly selected a sub-sample (20%) of transcripts to review, and an average percent agreeance of 81% was calculated to ensure sufficient inter-coder reliability and transparency within the coding process [26]. All coding discrepancies were discussed among FA and CR and agreed upon.

## Results

### Sample characteristics

A total of *N* = 45 participants were enrolled in the study. The average age of participants was 39.2 (± 9.1) and the majority identified as male (*n* = 24, 53%) and White (*n* = 27, 60%). Two-thirds of participants were stably housed (*n* = 29, 64%), however, *n* = 16 (36%) indicated they were homeless and/or unstably housed. See Table 1 for a breakdown of the sample characteristics.

**Table 1:** Self-reported study participants’ demographic characteristics

Demographic Characteristics	Total (*N* = 45)	Percentage (%)
Age Groups		
18–30	11	24
31–50	26	58
≥ 51	8	18
Sex		
Male	24	53
Female	20	45
Non-binary	1	2
Ethnicity		
White	27	60
Indigenous	17	38
Black	1	2
On OAT or safe supply^a^		
Yes	24	55
No	21	45
Living Situation^b^		
Stably Housed	29	64
Unstably Housed	8	18
Homeless	8	18
Location^c^		
Rural/ Remote	4	9
Urban	41	91

^a^Safe supply included those who were receiving drugs (either opioids or stimulants) though a prescription program

^b^Individuals indicated whether they considered their current living situation as stable or unstable

^c^Individuals indicated whether they considered the geographical location of their living situation as either rural/remote or urban

Regarding substance use patterns, participants represented diverse PWUD with varying substance use profiles and lifestyles (Table 2). Many participants, (*n* = 24, 53%) identified as polysubstance users, and over three-fourths (*n* = 35, 79%) indicated they used illegal substances daily.

**Table 2:** Study participants’ substance use characteristics

Substance Use	Total (*N* = 45)	Percentage (%)
Substances		
Polysubstance^a^	24	53
Stimulants	9	20
Opioids	12	27
Frequency of Use		
Daily	35	78
Weekly	10	22

^a^‘Polysubstance’ use included reference to using two or more categories of substances, primarily opioids and stimulants, as well as using speedballs (a combination of stimulants and opioids). ‘Stimulants’ primarily included uppers such as cocaine, crack-cocaine and amphetamines including methamphetamine/crystal meth; ‘Opioids’ primarily included downers including both illegal and pharmaceutical opioids such as hydromorphone, heroin and fentanyl, but excluded references to OAT such as buprenorphine with naloxone or methadone

### Qualitative results

Participants provided responses to questions regarding multiple aspects of the decriminalization policy, with the majority expressing strong opinions toward the 2.5g threshold. The results are outlined below and are narratively reported under the following two categories: 1) Implications for substance use profiles and purchasing patterns, including implications on the cumulative nature of the threshold and impacts on bulk purchasing, and 2) Implications of police enforcement, including distrust of police use of discretion, potential for net widening, and the potential for jurisdictional discrepancies in enforcing the threshold.

#### Implications for substance use profiles and purchasing patterns

##### Substance use profiles

Some participants suggested that the proposed threshold was appropriate for their daily personal use, and that they were unlikely to purchase or carry more than 2.5 g at a time. This view was particularly evident among participants that were unstably housed (e.g., individuals experiencing homelessness) or those who expressed that they typically could not afford to purchase more at a time. These participants often suggested that the policy would therefore not likely affect their use patterns. For instance, the following participant suggested that the threshold would likely be appropriate for marginalized populations or individuals who cannot afford to purchase large amounts of substances at a time:

*“I think it’s okay for people who are living a minute at a time, surviving a minute at a time to their next hit, who can’t afford to buy larger and spread it out, just because they’re so marginalized with poverty and don’t have the means. They’re just trying to survive and support their habit. So when somebody’s put in that situation they’re literally surviving...” (Female, age 40, Indigenous)*

However, the majority of participants expressed discontent with the 2.5g threshold, suggesting it is far too low, and that it does not reflect their substance use patterns. This was particularly the case among those who participated in high-frequency substance use: *“This 2.5 g, I could do that before noon” (Male, age 41, Indigenous).* High-frequency and large-quantity use patterns were commonly reflected among polysubstance users who often indicated they had high drug use tolerances and therefore needed to use more at a time:

*“I think it needs to be up to whatever certain amount, I’m not sure exactly, but some people, like a person like myself, we [me and my partner] both have extremely high metabolisms and we have extremely high drug tolerances. Especially myself, when it comes to opiates, I’m on 253 mls of Metadol, fentanyl, 10 [Dilaudids] a day, plus, you know what I mean?” (Female, age 44, White)*

Here, the participant emphasizes that individuals’ drug use and tolerance levels are diverse, and suggests that even among those who are engaged in opioid agonist treatment and/or safe supply prescription programs, the 2.5g threshold is still not high enough. Overall, participants expressed that the 2.5g threshold was not conducive to their substance use patterns and frequency of use and was particularly not reflective of polysubstance users or those with higher tolerances.

#### Implications of the cumulative threshold on purchasing

In addition to participants indicating that the 2.5g threshold would not be reflective of their use patterns, they also indicated that it was not reflective of their purchasing patterns. Many participants—and particularly polysubstance users—suggested that they usually buy different amounts for each substance they consume and carry them all on their person at one time: “*A lot of people have more than one on them, like a lot of heroin users have meth. So I don’t think [the 2.5 g threshold is] enough” (Female, Age 44, Indigenous).* As such, participants suggested that the cumulative nature of the 2.5g threshold would not apply to them since they purchased multiple drugs and amounts at a time: *“The thing I probably have the strongest opinion on, is they shouldn’t lump all the drugs into one group” (Male, age 50).*

Statements by participants on the ‘arbitrary’ nature of the 2.5g threshold underscored confusion regarding how policymakers came to that number and reinforced how people who use drugs’ voices and realities are commonly excluded or disregarded when it comes to implementing policies that impact their lives.

Participants also noted that there are significant differences in the types of substances and how they are commonly sold. For instance, it was suggested that the 2.5g threshold may be more appropriate for specific substances such as heroin or fentanyl, which participants stated they often purchase smaller amounts of (usually in points [one tenth of a gram] instead of grams). Whereas other substances, such as methamphetamine or cocaine, were usually packaged and sold in larger quantities. Based on this, some participants who were polysubstance users suggested that they would likely still carry more than the threshold amount at any given time, leaving them vulnerable to criminalization under the threshold:

*“I do crystal meth, and normally I pick up more than that for personal use every time when I do it. So I would be over that threshold right away if I got stopped. So that law would be kind of useless in a way…but for down (the colloquial term for fentanyl-derived street drugs), I think I would be okay with that amount. But for the uppers (stimulant-derived drugs) I think it would be not enough…2.5 grams, isn’t a huge amount. Especially when you’re looking at drugs like crystal meth, like that’s not much for cocaine. That’s a very very small amount. With street down, it’s good for that one drug I think.” (Male, age 41, White)*

Many participants therefore suggested that it would make more sense for the threshold to be either substance-specific (i.e., not cumulative), as it is in other jurisdictions that have decriminalized illegal substance use, or be a set amount that is commonly purchased, such as 3.5 g (colloquially referred to as an ‘8-ball’):

*“It’s a weird number that they chose because it’s not an amount most people buy? It’s an amount that just seems really arbitrary because, like, it doesn’t make sense why they chose 2.5 grams because a lot of people buy 8-balls, which is 3.5 grams. It just seems really strange that they chose 2.5 grams.” (Male, age 30, Indigenous)*

#### Implications for bulk purchasing

Another implication related to purchasing patterns was the need to buy in bulk. Many participants suggested this practice as an economic strategy to save on costs considering that the more they purchased at a time, the less it would cost them. For instance, one participant described the cost savings they received when they purchased their substances in bulk, which, under the current threshold, would render them vulnerable to criminalization:

*“I’d like to see a higher amount [threshold] because I know for myself whenever I get a check I always try to buy in bulk. In Prince George one-point costs anywhere from $20-$40 dollars, where a half gram cost $50-$70 dollars, and one gram is from $80-$120 dollars, and then a half ball is usually $150 dollars. The prices decrease so drastically when you buy more. By the time you get a quarter (7 grams) you’re only paying $700. When I buy that amount, it’s for personal use.” (Female, age 42, White)*

Purchasing in bulk as an economic strategy was particularly endorsed by participants who resided in smaller or Northern communities:

*“I can see 2.5 grams being a starting point but that’s not the whole category of people who use drugs, and most people who use drugs, especially for more spread-out areas like rural, remote, it’s going to be people buying larger quantities getting the best value for their dollar and trying to ration it or use it as needed.” (Female, age 40, Indigenous)*

Participants who resided in these areas and communities indicated that it is difficult to commute/travel to get their substances and that they would often send one person to an urban setting to collect their drugs on behalf of a few people. Participants cited poor weather conditions, affordability (including the need for people to pool their money together), or to reduce the amount of travel time, as justifications for this practice:

*“People living in the middle of nowhere sometimes drive for two days to get their supply. So, for someone to be able to access enough to get through half a week, or a week, would make more sense.” (Male, age 50, White)*

Consequently, participants suggested that the threshold limit was too low, especially among those residing in more remote locations.

Participants also described how they often engaged in practices of sharing and/or splitting their substances among their peer networks or significant others to make purchasing substances more affordable, and reflected on the implications of this practice under the new policy:

*“[People] buy in large amounts for different reasons, either they pool their money together or they don’t wanna look suspicious leaving their house multiple times and stuff, and so the threshold amount would impact them.” (Male, age 30, Indigenous)*

Many participants suggested that if they were to abide by the 2.5g threshold to avoid criminalization, they would end up spending more money than they could afford: *“I would use just under 2 g, a half ball a day. So that means I would have to purchase every day in order to keep under the laws, which is more expensive.” (Male, age 52, White).*

Other justifications for purchasing in bulk included purchasing from trusted dealers/sources. Participants often described having go-to sources that they relied on as a pseudo- ‘safe supply’ strategy, particularly when the dealers tested their substances in advance. Participants also suggested that having to purchase from sources they did not trust meant that they were more likely to acquire adulterated/toxic substances since they could not be certain the dealer had tested the drugs prior. Participants described that having to adhere to the 2.5g threshold would force them to purchase more frequently, which could therefore result in increased exposure to risks associated with use, as well as to increased police surveillance:

*“For me, for instance, I buy seven [grams] at a time. It’s the most economically friendly. I get higher quality because it hasn’t been mixed yet into different smaller quantities. And it also decreases contact risk. So going out and meeting someone, I only have to do that once a week, whereas if I was gonna stay with 2.5 grams, I’d probably have to go out every day or at least every other day.” (Female, age 33, White)*

Other participants also elaborated on this point and described confidence in purchasing larger quantities of substances from dealers since they considered it to be safer as the drugs were less likely to be ‘stomped’ or ‘buffed’ with adulterants, like fentanyl, which was common practice for smaller quantities:

*“Most of the time you see methamphetamine [in the fentanyl] it’s because of cross-contamination. The guy cuts up an ounce of methamphetamine and uses the same scale to measure up half an ounce of fentanyl…you’re getting cross-contamination. (Male, age 66)*

Overall, participants suggested that the 2.5g threshold has substantial implications on participants’ purchasing patterns. These implications may be particularly detrimental for people who use drugs who need to purchase larger quantities at a time due to polysubstance use, high drug use tolerances, for financial reasons, or to ensure access to what participants deemed to be a ‘safe supply’ from trusted dealers who test the product before selling it.

#### Implications of police enforcement

##### Distrust of police use of discretion

In addition to the implications of the threshold itself, participants alluded to systemic issues of distrust against police, and suggested that police discretion would play a large role in the application and enforcement of the 2.5g threshold. They believed that the role of policing would continue to result in negative impacts that may undermine the objectives of the decriminalization policy. In suggesting the historical impact of police discretion, participants provided anecdotes of some of the ways in which they felt the police had previously abused their discretionary powers:

*“There’s cops out there that are real jerks who would love to bust anybody with the smallest amount, just so they make them wait all week. Like bust them on a Friday and make them sit in cells detoxing all week, which could send someone into acute withdrawal and be close to dying.” (Male, age 41, White)*

Many participants expressed cynicism towards the policing and justice system based on negative past experiences. Participants thus inferred that police would likely continue to use their discretion inconsistently under the new policy, and that interactions and consequences would depend on individual police decision-making:

*“I believe it depends on what side of the bed the police woke up on that morning. I see people getting harassed for carrying and then the next day the police walk by them on the street when they’re clearly using. So there’s absolutely zero consistency and I think it’s all a very personal decision dependent on who’s involved.” (Female, age 51, White)*

#### Potential for net widening

Participants further proposed a number of potential unintended consequences related to police use of discretion. For instance, some participants suggested that with a defined threshold quantity, the police may be more inclined to target and arrest people who use drugs who are carrying amounts slightly above the threshold, and charge them with either possession for personal use or for trafficking. In other words, participants were fearful that the police would use the threshold amount as justification to approach people who use drugs to ‘check’ that they were carrying under the threshold, which could result in increased consequences for those who were caught carrying more on them: *“I can see this being a problem as well. I can see giving cops opportunities to harass and pull over people just to make sure they’ve got a little bit over 2.5 g” (Female, age 40, Indigenous).* Participants suggested the potential for a net-widening effect based on the 2.5-g threshold, where individuals who may not have been stopped or arrested prior to decriminalization may now be criminalized:

*“It makes me feel really shitty. It makes me sad. They know that’s not enough and the thing is that people have to carry more than that [2.5 grams]. So what, now are they gonna have rights to probable cause to arrest anybody who they think uses drugs to make sure they’re only carrying their allotted amount of a decriminalized supply?” (Female, age 44, White)*

The potential net-widening effect was a specific concern for participants who suggested it would disproportionately impact the dealers and suppliers who they trusted to provide them with an unadulterated supply. Their fear was that police officers may start targeting drug trafficking which would result in increased risk of overdose and harms for people who use drugs, if their trusted dealers were arrested:

*“Who I worry about is the dealers, and if I have a dealer that I’ve established a relationship with and I know his stuff, and he knows me and what I like, and we’ve got a really good rapport and I’ve got some consistency finally, that’s actually keeping me safe. But if he gets busted and goes to jail, then somebody else is going to take his place. And generally, what ends up happening is that it’s somebody who doesn’t really know what they’re doing, doesn’t have the same experience, isn’t going to have the same quality supply that I’m used to. Probably there’s gonna be more weird dope floating around for a while, like there was early on in the pandemic. It’s just more dangerous…That’s what I worry about the most, I think it actually puts us at more risk if our dealers are getting busted.” (Female, age 37, White)*

Given the potential for increased criminalization of dealers, some participants who identified as both users and low-level/survival dealers expressed concern that they may face increased scrutiny under the decriminalization policy:

*“Most people who are selling are selling out of necessity themselves. It’s the only career they know, it’s the only thing they know. It’s comfortable to them. And they’re doing something that most people don’t do, which is actually testing their stuff and caring about what’s in it. It’s an exceptional case but it still makes me mad that they would be at risk for criminal charges.” (Female, age 33, White)*

#### Potential for jurisdictional discrepancies

Regarding the potential for inconsistent application and enforcement of the 2.5g threshold, participants suggested that smaller, Northern, or isolated communities could be particularly vulnerable to police use of discretion, which could result in important jurisdictional disparities. For instance, many participants believed that police in rural and remote settings were more likely to criminalize and discriminate against people who use drugs. Additionally, participants suggested there may be stark differences in policing culture, ideologies, and practices between urban and rural police departments. Many participants proposed that police officers in larger, urban police departments, such as the Vancouver City Police Department (VPD), were more likely to ignore personal possession of small amounts of drugs compared to smaller or rural areas. Specifically, participants suggested that urban police forces, such as those within Vancouver in particular, may already have been practicing de facto decriminalization and not charging people who use drugs for personal possession in high-traffic drug use areas:

*“Downtown Eastside (Vancouver), the small amounts of dope have pretty well been legalized anyway. But again it’s the arbitrary. All drug laws are arbitrary. You can have two people standing next to each other, both have dope on them, the police know and is gonna arrest the fucker he don’t like.” (Male, age 66, White)*

As such, participants suggested that the impact of the decriminalization policy, as well as the degree to which the 2.5-g threshold is enforced, will likely differ based on the community:

*“If you have under three grams they don’t even look at you, they don’t even bother. We have way bigger fish to fry than some personal [possession]. So like, in Vancouver, it’s sort of a known thing that they just don’t. But say in Nanaimo (a more rural community), they will bust you for like a point. They’re literally out to criminalize drug users and homeless people, because that’s what they do there. So it depends on the community.” (Female, age 44, Indigenous)*

Overall, participants expressed fears and concerns around the potential impacts of police enforcement of the 2.5g threshold, including the potential for net widening as well as for important jurisdictional differences. Participants suggested a deep-rooted distrust of the police and believed police discretion and enforcement would likely result in unintended negative consequences.

## Discussion

BC’s decriminalization policy presents an opportunity to shift the policy landscape of drug use towards a public health framework, recognizing the vast positive impacts the policy can offer if it can successfully fulfill its objectives of reducing stigmatization, increasing access to health and social services, and ultimately reducing the public health burden of the overdose crisis. The responses gleaned from participants in this study provided key insights on factors that should be closely monitored when evaluating the impacts of the 2.5g threshold, including in relation to substance use and purchasing patterns, as well as the application of police discretion in implementing the policy.

The study’s findings emphasize that although some participants expect decriminalization to result in positive outcomes and felt as though the 2.5g threshold was appropriate, the majority of participants foresaw a number of significant limitations due to the defined threshold quantity. Our findings offer insights into what those limitations are. This is in line with the research and consultation process that was conducted by BC’s Ministry of Mental Health and Addiction to inform the exemption request, and by the many advocates who continue to recommend a higher threshold limit that more accurately reflects people who use drugs’ substance use profiles in BC [20]. Participants in our study proposed a number of factors that may undermine the effectiveness of the 2.5g threshold, such as continued need to purchase substances in smaller quantities, which has the potential to be “stomped” or contaminated with other substances, thus potentially increasing overdose risk. Additionally, with a threshold limit so low, it could create a market for substances to become more adulterated, which could make them increasingly dangerous for people to use. As research in other jurisdictions has shown, drug policy interventions that target drug markets can have severe impacts on the safety of the drug market and can increase overdose risk and other harms for people who rely on it [28–30]. People in our study who relied on purchasing drugs in bulk suggested that the threshold could result in additional financial costs and increased overdose risk. As well, police discretion to arrest and charge *above* the 2.5g threshold could result in the unintended consequence of *increasing* drug-related arrests, such as through targeted search and seizures and increased surveillance of drug trafficking.

The implementation and enforcement of the policy, and particularly the 2.5g threshold, will likely be of utmost importance when evaluating whether the policy is meeting its proposed objectives, as the threshold will be used to delineate between those who will be criminalized versus those who will not. Currently, there is no publicly available information regarding what types of information police will take into consideration when deciding what amount above the 2.5g threshold will be considered possession for personal use versus for trafficking purposes, and whether a criminal or health response will be taken. This therefore has significant implications for law enforcement who are tasked with enforcing the policy. Data from Australia suggest that based on individual drug use patterns, even when there are clear threshold limits for personal possession/use versus trafficking, some people who use drugs are still at risk of being criminalized for possession and/or trafficking if their personal use exceeds current thresholds [16]. Recognizing this, it has been suggested that in BC, the threshold should be considered a ‘floor’ not a ‘ceiling’ [19], meaning that people who possess over the 2.5 g threshold should not automatically be considered as carrying for trafficking purposes and that law enforcement should be guided by explicit direction to avoid criminalizing people who use drugs. Such a broad interpretation would recognize that people who use drugs who have varying patterns of use might need to possess over the 2.5g limit but would not necessarily be doing so for trafficking purposes.

Our study also underscores the importance of recognizing the long history of uncertainty, punitive actions, and negative experiences with police among people who use drugs. Decriminalization in BC and implementation among police has important implications regarding building trust between people who use drugs, the community, and law enforcement. While there is the potential to reduce stigmatization and criminalization against people who use drugs, the discretionary power of law enforcement will play a large role in achieving these outcomes. Given the fear of police discretion and subsequent criminalization, the enforcement of the 2.5g threshold by police will be pivotal in reducing criminal penalties for people who use drugs in BC. Participants feared that some cities, particularly rural and remote or Northern and more isolated locations, would still experience criminalization for their drug use, and this was especially noted for marginalized and racialized populations and rural/remote communities. These sentiments have been noted in previous qualitative research on decriminalization where people who use drugs in Australia expressed concerns about how discretionary practices by police would impact the ways in which the policy is implemented, and called for clearly defined law enforcement measures to eliminate any discrepancies or grey areas in enforcement [31]. In Canada, previous reforms to drug policy, such as the Good Samaritan Drug Overdose Act, were ultimately undermined by a lack of knowledge and implementation among police, who continued to arrest individuals for possession despite the decriminalization of simple possession at overdose events [32, 33]. This policy had a number of shortcomings, such as ambiguity around police’s discretion when encountering people with drug paraphernalia on them or those who had outstanding warrants for their arrest, demonstrating the importance of recognizing the potential harms that shortsighted policies combined with continued use of police discretion may result in.

Although study participants, people who use drugs and their allies have called for a more hands-off approach and an overall decentralization of police involvement in drug use, citing major concerns in relation to police use of discretion, as it stands, the policy and the 2.5g threshold will continue to be enforced by police [34]. Therefore, police knowledge on decriminalization and its goals, as well as training, will likely play a direct role in how police apply their discretion during enforcement of the policy. As part of the policy implementation plan, the BC MMHA have incorporated different phases of robust police training starting with Phase 1 in November 2022, and Phase 2 launching in Summer 2023 [35]. While the specifics of the training modules are not publicly available yet, the implementation paths allude to the importance and need for tailored and targeted police training measures. These training measures should incorporate awareness and education on different substance use practices and profiles that may criminalize people who use drugs who are polysubstance users, or who carry more than the allocated threshold because of location, tolerance, need, or accessibility. Frontline law enforcement officers must be made aware of established service pathways to be able to support people who use drugs, and adjunct health system improvements will need to be implemented to strengthen these connections and the capacity of services to provide support. Furthermore, under the MMHA plan, it is imperative that appropriate resources, training, and education are provided to inform police on how to engage with people who use drugs from different communities, guided by a public health and anti-stigma lens. If law enforcement officers are trained on how to identify different drugs, the ways in which drugs are commonly sold and packaged, and the various patterns of use among polysubstance users, then they may be able to exercise more appropriate discretion when applying the 2.5g threshold during an interaction with people who use drugs. As part of the policy, police will be mandated to provide resource cards with information on local health and social services to people who use drugs who request them, and provide referrals to these organizations upon request [35]. These connections will also be key to the policy’s objectives, and if done correctly and appropriately, can reduce stigma, and facilitate access to treatment or harm reduction services [36]. However, extant research suggests that even in situations where police have de-penalized simple possession, the ways in which this is enforced can vary and can result in significant inconsistencies, inequities, and harms, including net widening effects [37].

Overall, participants expressed that decriminalization is undoubtedly a positive step forward in addressing the overdose crisis. Based on data from the European Monitoring Centre for Drugs and Drug Addiction, following decriminalization, Portugal experienced a significant reduction in mortality among people who use drugs, particularly in the first several years after policy implementation [38, 39]. Furthermore, the prevalence of drug use in Portugal consistently remains below the European average and treatment uptake has increased significantly, and the policy has been one of the most influential in prompting drug reforms in other countries [40, 41]. This data underscores the potential public health benefits of decriminalization. However, participants in our study suggested that *“it is only one tool in the toolbox”.* The policy requires close and rigorous evaluation to understand how its implementation will impact the lives of people who use drugs and their risk of harms, including morbidity and mortality. Understanding the ongoing factors associated with the realities of people who use drugs and the reasons as to why they may carry a range of drug amounts, both under and above the 2.5g threshold, can help ensure the decriminalization policy is implemented in an effective manner that is reflective of their realities.

Limitations of the current study must be noted. Although we made our best efforts to include participants from a wide variety of backgrounds, geographic locations, and substance use patterns and experiences, we recognize the participants do not represent all people who use drugs in BC. Due to our recruitment strategies, they may be biased towards those who are more integrated and connected with the harm reduction services and advocacy groups through which study recruitment occurred. As such, the data may not be generalizable outside of the specific contexts and from the participants they were collected. Moreover, in their responses, some participants tended to generalize and reflect on the community of drug users as a whole, and not specifically on their own use or direct impacts of the policy on their own lives, making it difficult to decipher the specific impacts of the policy on individuals versus on the whole community at large. However, we are confident that the responses reflect diverse perspectives on the potential implications of the 2.5g threshold limit. While self-reported demographic data was collected, the present study did not disaggregate this data, However, these factors are important to consider in understanding the impact of the threshold on gender, age and ethnicity, and these dynamics should be explored through future research.

## Conclusion

As BC has just implemented a monumental shift in drug policy, from decades of criminalization to decriminalization with a public health lens, the proposed threshold limit and how this is implemented and enforced by law enforcement will have substantial impacts on people who use drugs. This will play a huge role and will dictate whether the policy is a success. Participants interviewed in this study indicated that the 2.5g threshold limit as it stands may increase their risk of drug-related harms such as overdose and arrest. As the policy unfolds, it will be vital to monitor and evaluate the impacts of the threshold to ensure it does not result in further harms to people who use drugs.

## Data Availability

Not applicable.

## Abbreviations

BC: British Columbia
PWUD: People Who Use Drugs
CDSA: Controlled Drugs and Substance Act (CDSA)
MMHA: Ministry of Mental Health and Addiction
CRISM: Canadian Research Initiative in Substance Misuse
OAT: Opioid Agonist Treatment
CAMH: Centre for Addiction and Mental Health
